# Descemet stripping endothelial keratoplasty for a failed penetrating keratoplasty graft in a pseudophakic patient with a toric intraocular lens: a case report

**DOI:** 10.1186/1471-2415-13-64

**Published:** 2013-10-30

**Authors:** George D Kymionis, George A Kontadakis

**Affiliations:** 1 University Hospital of Heraklion, University of Crete, Medical School, 71003, Heraklion, Crete, Greece; 2 Bascom Palmer Eye Institute, University of Miami, Miami, USA; 3 Ophthalmiatreio Eye Hospital of Athens, Athens, Greece

**Keywords:** Descemet stripping automated endothelial keratoplasty, Post penetrating keratoplasty astigmatism, Toric intraocular lens

## Abstract

**Background:**

To report a patient with penetrating keratoplasty (PKP) graft endothelial failure implanted with toric intraocular lens (IOL) who was treated with Descemet stripping endothelial keratoplasty (DSAEK).

**Case presentation:**

A 40 year old male patient implanted with toric intraocular lens for the treatment of post PKP astigmatism, presented for the treatment of graft endothelial failure’. The patient had uncorrected distance visual acuity (UDVA) 20/200 not correcting with manifest refraction. The patient reported excellent visual acuity after cataract surgery and toric IOL implantation. DSAEK was performed in order to minimally affect keratometry and retain correspondence of the anterior cornea astigmatism with the toric IOL astigmatic power. Three months postoperatively the cornea was clear with no edema. UDVA was 20/40 and corrected distance visual acuity was 20/25 with +1.50-1.00 × 20.

**Conclusions:**

This report describes a unique case of DSAEK for treatment of a failed PKP in a patient previously implanted with a toric IOL. DSAEK was an effective alternative of PKP in this patient for the preservation of the toric IOL’s effect.

## Background

Endothelial dysfunction is one of the leading causes for visual impairment of corneal etiology. Until recently penetrating keratoplasty (PKP) was the primary treatment for corneal endothelial disease [1]. Descemet stripping automated endothelial keratoplasty (DSAEK) is a less invasive technique for the treatment of corneal endothelial disorders with fewer intraoperative and postoperative complications than PKP [1,2]. The use of DSAEK as an alternative for repeat PKP after graft endothelial failure has been already reported with excellent results especially when there is no high post PKP astigmatism in the graft [3,4].

Treatment of postoperative astigmatism has always been one of the most significant considerations after PKP. Several methods have been described for the management of post-PKP astigmatism, such as limbal relaxing incisions, excimer laser refractive surgery, arcuate keratotomy, and toric phakic intraocular lenses (IOLs) [5]. Cataract extraction and implantation of toric IOLs has also been reported as an effective alternative even for high astigmatism [5-7]. A significant consideration is the mismatch in toric IOLs astigmatic power with the corneal astigmatism in cases were repeat PKP is needed after toric IOL implantation.

We present a case of a patient who was implanted with a toric IOL to treat post-PKP astigmatism and shortly afterwards developed graft endothelial failure. The patient was treated with DSAEK.

## Case presentation

A 40 year old male presented to our institute with PKP graft endothelial failure. The patient had undergone primary PKP 10 years ago due to keratoconus. The patient had developed cataract one year ago. He underwent cataract extraction and toric IOL implantation in order to treat cataract and astigmatism simultaneously. He reported excellent visual acuity postoperatively. Six months after cataract surgery the patient experienced a graft rejection episode that was treated unsuccessfully and lead to graft endothelial failure.

**Figure 1:**
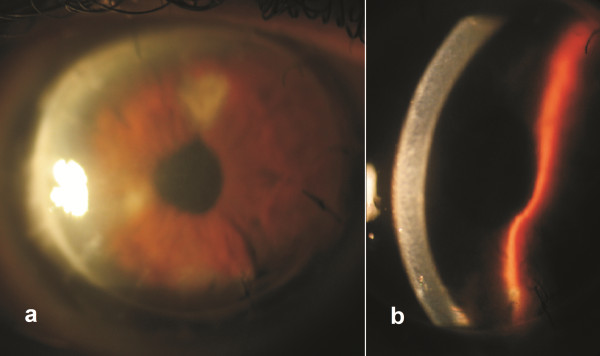
**Patient at presentation.** Slit lamp photographs of the patient at presentation **(a)** with broad illumination, and **(b)** slit illumination. Significant edema is evident.

At presentation, his uncorrected distance visual acuity (UDVA) was 20/200 and his best corrected visual acuity was 20/200 not correcting with manifest refraction. Slit lamp evaluation revealed significant corneal edema (Figure 1) and corneal topography could not be performed due to epithelial edema and irregularity. According to data provided by the patient, he was implanted with an Alcon AcrySof Natural SN60T7 IOL (Alcon Laboratories, Fort Worth TX, USA).

In order to avoid the complications of repeat PKP and to minimize any change is postoperative astigmatism, we decided to perform DSAEK. The patient was informed and signed his consent according to the institutional guidelines and in compliance with the Helsinki Declaration.

Pre-cut corneal graft was used for DSAEK. Flap diameter was 9.50 mm and graft thickness was 116 μm. The endothelial cell density was reported to be 3145 cells per mm [2]. The PKP graft had a diameter of 8.50 mm and the pre-cut graft was trephined to diameter of 8.00 mm in order to be smaller than the PKP graft and thus minimize possibility of graft detachment and of intraoperative PKP wound dehiscence [4].

Descemet’s membrane was stripped, to a previously determined size of 8.00 mm through a 2.6 mm clear corneal incision. The clear corneal incision was enlarged to 5 mm. The donor lenticule was loaded in a Busin glide and a micro incision forceps was passed across the anterior chamber through a side port, exiting through the enlarged clear cornea tunnel to grab the graft and drag it into the eye. The graft was then unfolded in the anterior chamber with a combination of balanced salt solution and air. Once the graft was open and centered, the anterior chamber was completely filled with air. At the end of procedure, a residual air bubble filling half of the anterior chamber volume was placed to support the graft postoperatively.

**Figure 2:**
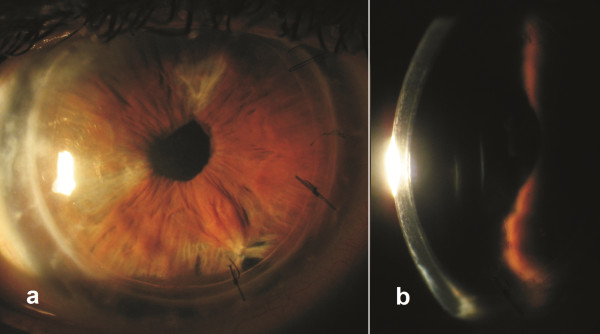
**The patient three months post DSAEK.** Slit lamp photographs off the patient three months post DSAEK **(a)** with broad illumination and **(b)** with slit illumination. Cornea is compact and clear, no edema either in the graft or in the host cornea is evident.

The patient’s postoperative course was uneventful. Postoperatively, the patient received topical antibiotics and cycloplegia for 1 week and topical dexamethasone 4 times per day for 1 month. After 1 month, the topical dexamethasone was tapered gradually over a 3-month period and finally discontinued. Three months postoperatively the graft was stable and the cornea was clear and compact with no edema (Figure 2). His keratometric astigmatism was 3.47 diopters. UDVA was 20/40 and corrected distance visual acuity was 20/25 with +1.50-1.00x20.

## Conclusion

Treatment of postoperative astigmatism in PKP patients is one of the largest concerns of PK surgeons [5]. It has been reported that 20% of patients have postoperative astigmatism more than 6.00 diopters [7]. Several methods have been proposed to treat this, such as laser refractive surgery, toric phakic IOLs, arcuate keratotomy and even repeat keratoplasty [5]. When there is also cataract, implantation of a toric IOL after cataract extraction has been reported to be a very successful treatment for even high degrees of astigmatism [5-7]. A significant concern in this technique is that if graft failure occurs in a patient implanted with a toric IOL and repeat PKP is performed, the postoperative corneal astigmatism is very likely not to compensate for the intraocular astigmatism of the IOL. Toric IOL misalignment with respect to corneal astigmatism, could lead to under correction or even induction of refractive astigmatism and hyperopic spherical shift [8,9]. If repeat PKP is to be performed in such patients, then the combination with IOL exchange and monofocal IOL implantation may be imperative for the refractive rehabilitation of the patient.

DSAEK is considered to be a less invasive treatment for endothelial dysfunction in comparison to PKP, that offers patients faster visual rehabilitation, good visual results, and stable topography [1,2]. With this technique several possible complications of PKP are avoided, such as the increased risk for intraoperative suprachoroidal hemorrhage, the risk for postoperative wound dehiscence, the postoperative high astigmatism and the suture related complications [1]. In recent years DSAEK has been used for the treatment of endothelial dysfunction in several corneal diseases, such as Fuch’s dystrophy, bullous keratopathy, and Chandler’s syndrome [1,2,10].

Lately, the use of DSAEK as an alternative for repeat PKP after graft endothelial failure has been also reported with excellent results [3,4]. These patients are offered the opportunity to undergo regrafting with a less invasive treatment and less induced astigmatism. Especially in cases where postoperative astigmatism in the PKP graft is acceptable, regrafting with DSAEK is the preferred method.

In our case, in order to retain the preoperative association of corneal astigmatism with the implanted toric IOL, DSAEK was used as an alternative to repeat PK for the treatment of the patient’s failed PKP graft. Although we did not have data on the patient’s corneal astigmatism due to the inability to perform topography on the edematous graft, we decided to proceed with DSAEK based on the patient’s report of a post –cataract excellent uncorrected visual acuity in order to induce minimal change to the patient’s corneal astigmatism. Postoperatively, the patient still had low refractive astigmatism despite the mild corneal astigmatism, thus indicating that the toric IOL still compensated for the corneal astigmatism after the DSAEK.

In another case in the literature, Scorcia et al. reported implantation of a toric IOL at the same time as performing DSAEK [11]. The toric IOL in this case was implanted based on the preoperative astigmatism without knowing the effect that the DSAEK graft would have on the overall astigmatic error. The authors report excellent refractive results. Nevertheless, according to Terry et al. [2] the mean astigmatic change in a series of 100 eyes implanted with pre-cut DSAEK donor tissue was 0.09 diopters, thus justifying this intervention in order to maintain the compensating effect of the toric IOL on the corneal astigmatism.

In conclusion, DSAEK was an excellent alternative to repeat PKP in a patient previously implanted with a toric IOL. The fact that DSAEK minimally changed corneal astigmatism, allowed the preservation of the correspondence between the toric IOL’s astigmatic power and the corneal astigmatism, thus offering the patient a functional visual acuity.

### Consent

Written informed consent was obtained from the patient for publication of this case report and any accompanying images. A copy of the written consent is available for review by the Editor-in-Chief of this journal.

## Abbreviations

DSAEK: Descemet stripping automated endothelial keratoplasty; PKP: Penetrating keratoplasty; IOL: intraocular lens; DSAEK: Descemet stripping endothelial keratoplasty; UDVA: Uncorrected distance visual acuity.

## Competing interests

The authors have no financial or competing interests related to the materials or methods described in the manuscript.

## Authors’ contributions

GDK was the primary surgeon involved in the care of the patient and performed a critical review of the manuscript. GAK was involved in the care of the patient, performed the literature review and drafted the manuscript. Both authors read and approved the final manuscript.

## Pre-publication history

The pre-publication history for this paper can be accessed here:

http://www.biomedcentral.com/1471-2415/13/64/prepub
